# Pressure Equilibrium Time of a Cyclic-Olefin Copolymer

**DOI:** 10.3390/polym13142309

**Published:** 2021-07-14

**Authors:** Benedikt Roth, Dietmar Drummer

**Affiliations:** Institute of Polymer Technology, Friedrich-Alexander-Universität Erlangen-Nürnberg (FAU), Am Weichselgarten 9, 91058 Erlangen, Germany; dietmar.drummer@fau.de

**Keywords:** compression induced solidification, cyclic-olefin copolymers, equilibrium time, high-pressure capillary rheometer, counter pressure chamber

## Abstract

Integrative simulation techniques for predicting component properties, based on the conditions during processing, are becoming increasingly important. The calculation of orientations in injection molding, which, in addition to mechanical and optical properties, also affect the thermal shrinkage behavior, are modeled on the basis of measurements that cannot take into account the pressure driven flow processes, which cause the orientations during the holding pressure phase. Previous investigations with a high-pressure capillary rheometer (HPC) and closed counter pressure chamber (CPC) showed the significant effect of a dynamically applied pressure on the flow behavior, depending on the temperature and the underlying compression rate. At a constant compression rate, an effective pressure difference between the measuring chamber and the CPC was observed, which resulted in a stop of flow through the capillary referred to as dynamic compression induced solidification. In order to extend the material understanding to the moment after dynamic solidification, an equilibrium time, which is needed until the pressure signals equalize, was evaluated and investigated in terms of a pressure, temperature and a possible compression rate dependency in this study. The findings show an exponential increase of the determined equilibrium time as a function of the holding pressure level and a decrease of the equilibrium time with increasing temperature. In case of supercritical compression in the area of a dynamic solidification, a compression rate dependency of the determined equilibrium times is also found. The measurement results show a temperature-invariant behavior, which allows the derivation of a master curve, according to the superposition principle, to calculate the pressure equilibrium time as a function of the holding pressure and the temperature.

## 1. Introduction

Continuously shortening product life cycles and constantly increasing demands on product quality inevitably led to the use of injection molding simulations in the conception phase of the product development of polymer components. By calculating the filling processes of the complex geometries of an injection mold before the mold is manufactured, possible errors can be identified early on and time-consuming and cost-intensive iteration loops can be avoided [1]. The calculation processes are divided into a filling, holding pressure, and cooling phase. The filling process of a cavity can be resolved and predicted with sufficient accuracy using innovative mold designs [2,3,4] as well as increasingly detailed and modified material models [5,6].

Integrative simulation techniques will be used to locally determine the influence of the component geometry and the manufacturing process on the material properties [7]. For semi-crystalline materials, attempts are being made to model the factors influencing crystallization, such as shear-induced nucleation with superimposed cooling [8]. The aim of integrative simulations for amorphous polymers is, among other things, to predict the resulting optical, mechanical, and thermal component behavior on the basis of molecular orientations introduced during production [9].

In the injection molding process, the polymer chains are aligned in the direction of flow by applying an external shearing force, provided they are sufficiently mobile [10]. However, the macromolecules of a polymer melt strive for the energetically most favorable state of highest entropy in the form of a tangled cluster. The decrease in entropy and free volume associated with the orientation of the molecules results in an entropy-elastic restoring force, which transforms the polymer chains back into a thermodynamically more favorable state [11,12]. This process is known as relaxation and significantly determines the degree of orientation generated in the component during the manufacturing process and, thus, local anisotropies with respect to mechanical [13,14], optical [10,14], and shrinkage-related [15] properties. The speed of the intramolecular conformational ordering process depends on the mobility of the molecular chains and is thus determined by the free volume between the polymer chains of the amorphous melt [16]. As the free volume between the polymer chains increases with increasing temperature [17] and decreases with the orientation of the molecular chains [11], this affects the relaxation times. 

The free volume also shows a clear pressure dependence. This relationship is defined by the pvT-behavior of the polymer above the glass transition range [18]. A further indicator for the restriction of molecular mobility due to melt compression is the pressure dependence of viscosity. Here, the reduction of the space between the chains leads to an increased interaction of the polymer chains on the intramolecular level [19,20]. This is shown by an exponential increase in logarithmic viscosity with increasing pressure at low shear rates and temperatures near the glass transition [21].

Thus, the relaxation of the polymer chains, due to flow and high-pressure levels during the holding pressure phase, is prevented. The orientations introduced in the manufacturing process are primarily determined by the pressure, temperature, and shear conditions during the holding pressure and cooling phase [22]. During the filling phase, the orientations introduced can relax to a large extent, due to the shear-induced heating and low-pressure gradients at the melt front. In contrast, the orientations introduced in the holding pressure phase show a significantly lower relaxation rate, as a result of reduced mobility, due to the holding pressure level and the superimposed shearing as well as temperature decrease during cooling [23]. Pantani et al. was able to determine a significant increase in the molecular orientations with increasing holding pressure in injection-molded plates made of isotactic polypropylene and attributed this to an increase in relaxation times due to the high-pressure level [24].

In the literature, there are already several approaches to model and predict the molecular orientations of the resulting stress birefringence by viscoelastic material models. For example, Isayev et al. calculated the distribution of stress birefringence for injection-molded plates made of Polystyrene from flow-induced internal stresses using the Leonov model via the main equation of stress optics [25]. Flaman et al. extended this model to take into account the compressibility of the melt [23,26]. Lee et al. used the Leonov model to simulate the injection compression molding process, to predict flow-induced residual stresses and stress birefringence for radial polymer flow [27]. Pantani et al. used a nonlinear formulation of the elastic dumbbell model to describe the evolution of molecular orientations across the flow cross-section during the injection molding process [28]. Here, the relaxation time, as a function of pressure, temperature, and shear rate, is considered. The characterization of the relaxation times was performed with a rotational rheometer [29]. Furthermore, simulations have shown that the pressure dependence of the relaxation has a significant influence on the calculated orientations [24].

However, rotational viscometers are mainly used to supply a data basis for the calculation of the relaxation and orientation processes in injection molding simulation [29]. Deformation of an inactive melt under isothermal conditions, in a plate-and-plate configuration, and measurement of the applied torque to maintain this deformation over time generates absolute values for the relaxation behavior [30]. In this way the temperature dependence of the relaxation times could already be shown several times, whereby higher temperatures led to shorter relaxation times due to the increased free volume [31,32,33]. However, modeling the relaxation behavior on this basis does not seem to be practical for the injection molding process, where high-pressures gradients, and resulting flow dynamics, may also be acting during the holding pressure and cooling phase. Nevertheless, such investigations form the data basis for the calculation of the relaxation processes in simulations. In order to model the formation of orientations during the holding pressure phase, a better understanding of the pressure driven flow processes during the holding pressure phase is, therefore, indispensable.

The first investigations could already reveal a pressure dependence of the relaxation of orientations by means of simulations [34,35]. In addition, investigations using dielectric measurements to determine the dynamics of the chain segments show that the influence of pressure on the relaxation of orientations cannot be ignored [36]. Recent investigations by Reynolds et al. show a first approach to determine the pressure dependence of the relaxation of the molecular orientations, in the pressure range of 1–100 bar, as a function of the shear rate of a stationary flowing melt with a two-piston rheometer [37]. For this purpose, the capillary between the pistons was equipped with a quartz glass window, whereby the stress birefringence caused by the orientations could be visualized and its temporal development could be recorded and evaluated by a camera. The relaxation times measured from the decay of the fringes have been compared with relaxation times, determined from the decay of the pressure signals after the stop of both pistons. Within these investigations a pressure dependence of the relaxation time was found, but no shear rate dependence [37].

Previous investigations of the authors could determine a compression-rate-dependent, dynamic pressure-induced solidification in a high-pressure capillary rheometer (HPC) with a closed counterpressure chamber (CPC) at the capillary inlet and the pressure sensor during unsteady flow. The dynamic solidification has been characterized with polycarbonate (PC) by a shift of the solidification pressure to lower pressures with increasing compression rate under isothermal conditions. In addition, the measurement results showed the possibility of a temperature-invariant description of this solidification behavior [38].

With regard to the characterization of the formation and relaxation of orientations by polymer flow under process-relevant conditions and the question of whether a reflow of polymer through the capillary occurs after dynamic solidification, according to Roth et al., further investigations have now been carried out with the HPC and a closed CPC [38]. For this purpose, the dynamic pressure solidification behavior of a cyclic-olefin copolymer is first determined and a calculated master curve is used to derive an experimental design for investigating the temperature, pressure, and compression rate dependency of the flow processes after a dynamic solidification of the material. In order to quantify the velocity of the flow effects after dynamic solidification, the time courses of the pressure signals were observed and the completion of the flow processes was evaluated over a period of time, until a state of equilibrium was reached between all pressure signals.

## 2. Materials and Methods

A cyclic-olefin copolymer (COC) of the type 6013M (Topas Advanced Polymers GmbH, Frankfurt am Main, Germany) was used for the investigation. These are amorphous thermoplastics, which are frequently used in optical thin-wall components such as flat screens, light guided plates, and sensors due to their high-transparency and low-stress birefringence [39]. Due to its biocompatibility, the material is also becoming increasingly important in medical technology applications, such as micro fluidic chips. Since such thin-walled and micro components often show high-pressure gradients due to the increased surface-to-volume ratio during the filling and holding pressure phases, a characterization of the dynamic solidification behavior is of particular interest for this class of materials. According to the materials data sheet the glass transition temperature of the material is 142 °C.

**Figure 1:**
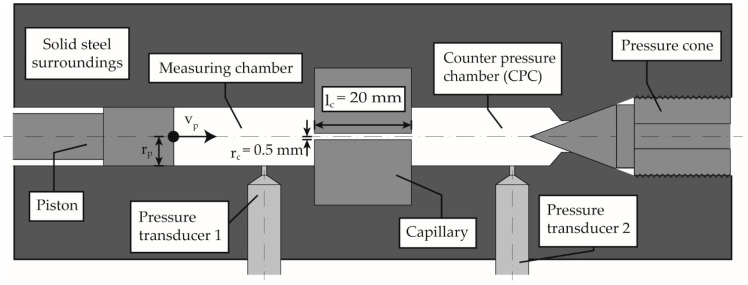
Measuring setup of the high-pressure capillary rheometer (HPC) with closed counter pressure chamber (CPC) according to [38]. Reprinted with permission from ref. [38]. Copyright 2020 Copyright Polymers.

The experiments were carried out on a HPC with a closed CPC (Rheograph 75, Goettfert Werkstoff Prüfmaschinen GmbH, Buchen, Germany) with a piston diameter of 15 mm. The measuring device used was specified with an accuracy of 0.4% for pressures of 20–2500 bar, whereby the maximum pressure corresponds to a piston force of 40 kN. To increase the accuracy, the elasticity and deformation of the frame, the drive train, and the force transducer are calculated and automatically corrected as a function of the piston force. Figure 1 shows the measuring setup schematically based on Roth et al. [38]. The permission has been obtained from the copyright holder.

First, the dynamic solidification behavior of the material has been characterized. For this purpose, the measuring chamber was heated to the set temperatures, then filled with polymer granulate in several steps, manually compressed, and degassed until the chamber was completely filled without air inclusions. To ensure the melting of the polymer, the piston was lowered onto the melt at a pressure of 2 MPa and kept at the corresponding temperature for 5 min. The molten polymer batch was then pressed through a built-in capillary into the CPC. After the whole system was filled with degassed polymer melt, the CPC was closed by screwing in the pressure cone, as shown in Figure 1. The temperature was maintained for another 10 min, followed by the corresponding compression. During compression at the different compression rates, the following data was recorded: piston position and speed, piston force and pressure at the pressure sensors (HDA 2174, Goettfert Werkstoff Prüfmaschinen GmbH, Buchen, Germany) in the measuring chamber p1 and in the CPC p2. The piston force was converted into the piston pressure pp, with the chamber cross section of 176.71 mm^2^. The capillary has a circular cross-section and the ratio of length to diameter of the capillary was 20/1. The methodology for determining the dynamic solidification pressures, as a function of temperature and compression rate, is described elsewhere [38]. A master curve was calculated for the material by measurements at the temperatures 210, 220, 230, 240, and 250 °C and the compression rates 1.8 × 10^−5^, 1.8 × 10^−4^ and 3.5 × 10^−4^ 1/s for the design of the experiments of the pressure equilibrium investigations. The compression rate *ψ* is calculated according to Equation (1):

(1) $$\psi =\frac{1}{V}\frac{dV}{dt}$$

where V is the total chamber volume and *d*V/*dt* is calculated from the product of piston velocity and piston area. The master curve was calculated according to the time-temperature superposition principle with a temperature shift factor α at a reference temperature T_0_ of 250 °C [40]. Table 1 gives an overview of the performed HPC/CPC experiments for the design of the experimental plan for the pressure equilibrium investigations.

**Figure 2:**
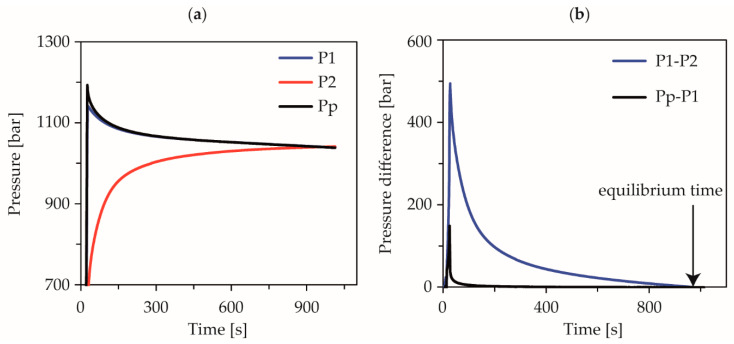
Course of the pressure signals after piston stop (**a**) and evaluation of the pressure difference between measuring chamber and counter-pressure chamber to determine the equilibrium time (**b**).

The measurement of the pressure-dependent equilibrium times was performed on the same measuring device under the same applied methodology of a closed CPC completely filled with melt. After compression at a defined isothermal temperature and underlying constant compression rate, the piston was locked and the pressure signals of the measuring chamber, CPC, and the piston force were recorded and evaluated at a frequency of 3 Hz. Figure 2a shows the behavior of the pressure signals after the piston has stopped, using the measurement at 240 °C, 1200 bar, holding pressure and a compression rate of 5.0 × 10^−3^ 1/s, as an example. The fast adjustment of the pressure signals of the piston force pp and the measuring chamber p1 to a pressure difference, which was recorded within the measuring accuracy of the pressure sensors, was interpreted as an elastic stress relaxation of the melt. The delayed approximation of the pressure signals of the measuring chamber p1 and the CPC p2 via the capillary was interpreted as pressure-induced equalization by viscous flow of the polymer. The calculation of the difference from the pressure signals p1-p2 allows the observation of a corresponding pressure equilibrium time, Figure 2b. The equalization process is completely finished as soon as this difference reaches the value 0. The time required to reach this state was measured as viscous equilibrium time in this study.

**Figure 3:**
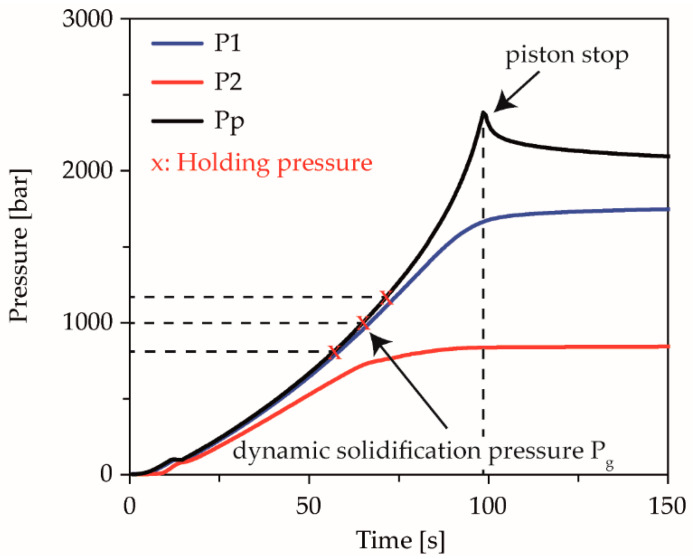
Illustration of the chosen holding pressure levels, calculated according to the master curve using the example of the performed limit experiment at 5.0 × 10^−3^ 1/s and 240 °C.

From the master curve of the preliminary tests, the dynamic solidification pressures for the respective temperatures were calculated under process related compression rates. In order to determine the influence of the compression rate, equal pressures with different compression rates were investigated for each temperature. By shifting the solidification pressure to lower pressures with increasing compression rate [38], an over-compressed state is obtained at the same pressure and a higher compression rate. The pressures were selected in such a way that according to the derived master curve at the lowest compression rate of 1.0 × 10^−3^ 1/s, the highest selected holding pressure corresponds to the calculated dynamic solidification pressure. At a mean compression rate of 5.0 × 10^−3^ 1/s, the mean selected pressure corresponds to the calculated dynamic solidification pressure, so that at the highest holding pressure an over-compression of 200 bar results (Figure 3). At the highest compression rate 1.0 × 10^−2^ 1/s, only the lowest holding pressure is below the dynamic solidification pressure, resulting in over-compression of 100 and 300 bar at the medium and the high holding pressure level. Thus, the influence of the compression rate on the equilibrium time can be resolved for still-fluid or dynamically solidified material during unsteady flow. To investigate the temperature influence, overlapping holding pressures were selected for each temperature.

The validation of the calculated dynamic solidification pressures at each temperature and compression rate was performed by experiments, up to the maximum load of the measuring device at 2500 bar. Figure 3 shows the pressure levels designed according to the master curve, exemplified by the experiment on the dynamic solidification behavior of the COC at a compression rate of 5.0 × 10^−3^ 1/s and an isothermal temperature of 240 °C.

Table 2 shows an overview of the examined temperatures, compression rates, and pressures. The pressures were varied for each temperature and compression rate given. In order to take into account the degradation of material during the measurements, the first measurement of the experimental plan (240 °C, 5.0 × 10^−3^ 1/s, 800 bar) was repeated at the end of the test day. In addition, the processed material was collected when the counter pressure chamber was emptied after the measurements and its viscosity number was compared with that of the virgin material. The viscosity number was determined according to DIN EN ISO 1628, with an Ubbelohde viscosimeter (0c, SI Analytics, Mainz, Germany), by dissolving the samples in a Decalin-Irganox solution with a concentration of 0.001 g/L for 45 min at 150 °C.

The influence of the heating of the melt as a result of compression has already been ruled out by extensive studies on this subject. For example, compression heating of a maximum of 2–3 K, at a slow compression speed of 16 bar/s, has been demonstrated for various amorphous thermoplastics at low compression speeds on the basis of several investigations [41,42,43]. The investigated cyclic-olefin-polymer was at the same level of about 3 K temperature increase when the melt tempered at 200 °C was compressed to 1000 bar at a compression speed of 16 bar/s, compared to a PC, which was also investigated [41]. In a further investigation by Rudolph et al., the influence of the compression speed and pressure level on the adiabatic temperature increase could also be determined for the investigated PC. Thus, at 260 °C melt temperature and compression with approx. 1600 bar/s, to a pressure level of 1600 bar, and a maximum heating of 10 K was measured [42]. Higher pressures and higher compression rates led to stronger compression heating of the melt. Since the compression rates in this study are a maximum of 200 bar/s and the maximum pressure level is 1600 bar, a possible error, due to excessive compression heating, has therefore been excluded.

## 3. Results and Discussion

### 3.1. Characterization of Dynamic Compression Induced Solidification

**Figure 4:**
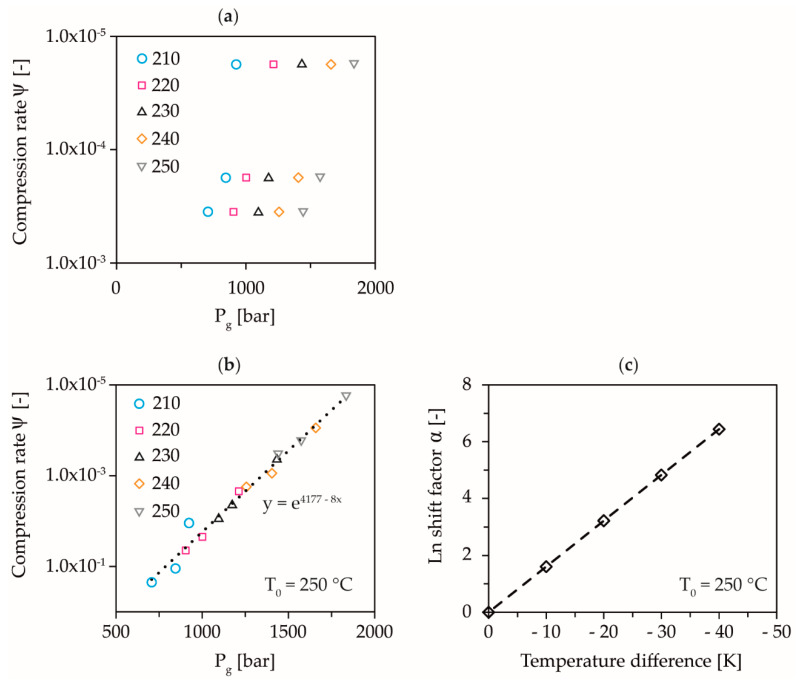
Determined dynamic pressure-induced solidification as a function of temperature and compression rate (**a**), shift of the dynamic pressure-induced solidification curves to a master curve at 250 °C (**b**), and temperature shift factors (**c**).

The results of the solidification pressures as a function of the compression rate determined, according to the methodology of [38] and the constructed master curve with the corresponding displacement factors for the COC, are shown in Figure 4.

An approximate linear decrease of the dynamic solidification pressure, with exponentially increasing compression rate is shown. In addition, higher pressures are required for the same compression rate and a higher temperature to dynamically solidify the material, Figure 4a. The free volume between the polymer chains is constantly reduced, due to the constant pressure increase in the initially compressible melt. Since the polymer flows simultaneously through the capillary due to the pressure differences, this requires a rearrangement of the polymer chains. Relaxation time is required for this, which increases with decreasing free volume, whereby the polymer cannot relax fast enough into a state of lower free volume when a certain pressure is reached and, therefore, behaves like a solid. The chains are then compressed only elastically, without any rearrangement processes taking place. The shifting of the curves according to the superposition principle over the compression rate with a temperature shift factor α allows the derivation of a temperature-invariant master curve of the behavior of dynamic solidification, Figure 4b,c. From the exponential fit of the master curve, an equation for the calculation of the dynamic solidification pressure as a function of compression rate and temperature can be derived:

(2) $$P_{g}=\frac{ln\left(\psi \right)-4.177-\alpha \times \Delta T}{-0.08}$$

**Figure 5:**
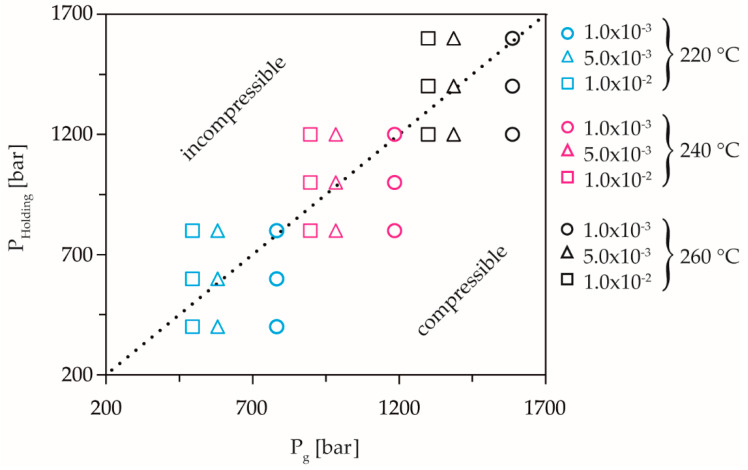
Depiction of the selected holding pressures in comparison to the calculated dynamic pressure-induced solidification pressures according to Roth et al. [38]. Reprinted with permission from ref. [38]. Copyright 2020 Copyright Polymers.

Using Equation (2), the solidification pressures for the compression rates and temperatures used in Table 2 could be calculated and the holding pressures for determining the equilibrium time could be designed accordingly. This is shown in Figure 5, where the experimental points on the original straight line correspond to dynamic solidification and all points above and below this line correspond to compressible or incompressible elastic compression of the melt.

**Figure 6:**
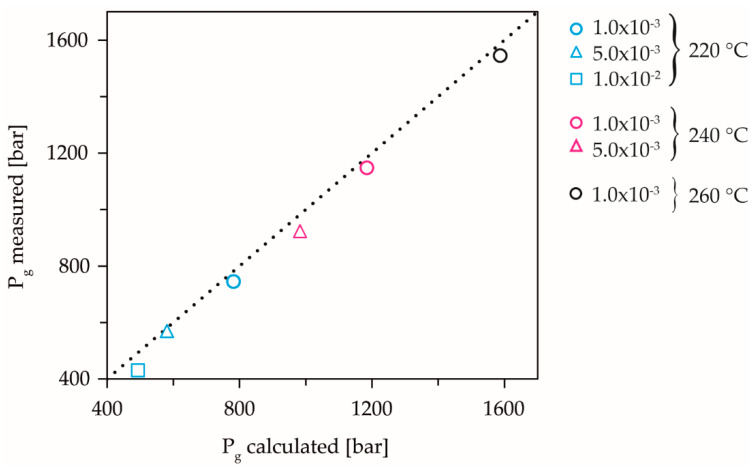
Validation of the calculation results by the performed limit experiments with evaluation according to Roth et al. [38]. Reprinted with permission from ref. [38]. Copyright 2020 Copyright Polymers.

The methodology according to Roth et al. does not allow a clear characterization of the dynamic solidification behavior at the selected application-oriented, high compression rates [38]. Nevertheless, the dynamic solidification pressures of the limit experiments could be determined for the low compression rates and, thus, a validation of the calculations from the master curve could be made. At the temperatures 240 °C and 260 °C and at the highest compression rate, the solidification pressures measured for validation of the master curve could not be determined exactly in the limit experiments, because the compression was too fast. However, all other measured solidification pressures show good agreement with the values calculated from the master curve, Figure 6.

### 3.2. Pressure-, Temperature- and Compression Rate- Dependent Equilibrium Time

**Figure 7:**
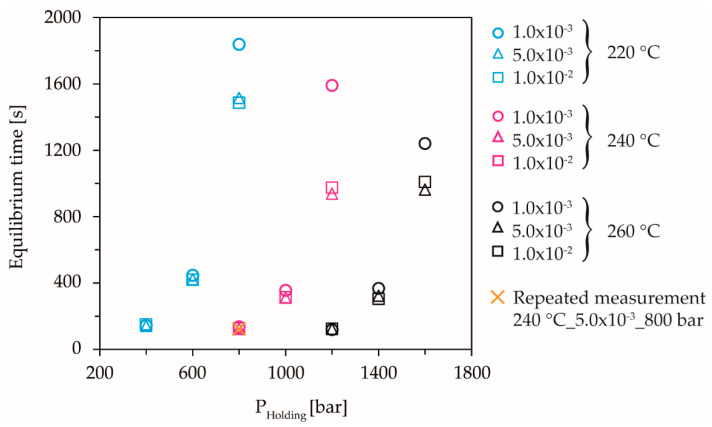
Measured equilibrium time as a function of holding pressure, temperature, and compression rate.

The results of the evaluation of the further above defined viscous equilibrium time are shown in Figure 7. The measured equilibrium times increase exponentially, with increasing holding pressure and decrease with increasing melt temperature.

The results are consistent with the presented model that increasing pressure and decreasing temperature cause an extension of the relaxation time due to the restriction of the intramolecular ordering process, caused by the lower free volume during unsteady flow. The compression-rate-dependence of dynamic solidification is thus also reflected in the equilibrium behavior. When applying the same holding pressure with a higher compression rate, the dynamic solidification pressure shifts to lower pressures. As long as the holding pressure is below the dynamic solidification pressure, there is no compression-rate-dependence of the equilibrium times. However, if the holding pressure exceeds the dynamic solidification pressure due to the compression rate-dependent shift of the dynamic solidification pressure, this results in an almost elastic compression of the melt in the measuring chamber. This leads to a higher-pressure difference between the measuring chamber and the CPC after the piston has stopped and, thus, to faster viscous equalization. The tests at 240 °C show the highest compression-rate-dependence. This non-linear behavior compared to the temperatures 220 °C and 260 °C is the subject of current investigations, in which the range between the measuring points at the highest holding pressure is to be resolved more finely by further compression rates. The repeated measurements at 240 °C, 5.0 × 10^−3^ 1/s, and 800 bar holding pressure ensured the reproducibility of the measurements and excluded the influence of aging. 

**Figure 8:**
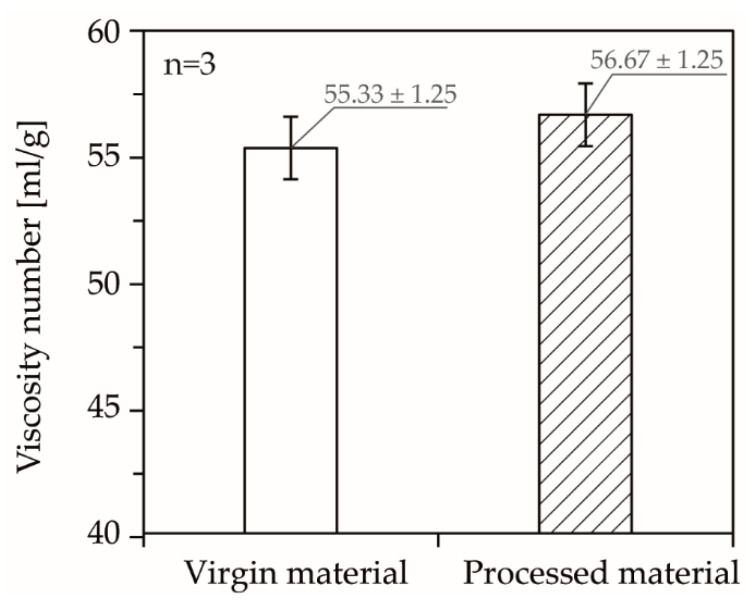
Comparison of the viscosity number of the processed material compared to the virgin material.

In addition, Figure 8 shows the measured viscosity numbers of the processed material compared to the virgin material. When taking into account the overlapping, standard deviations of the measurements, no significant change in viscosity number could be detected, which ruled out aging of the material during the 10 h test period. A low thermooxidative aging of the material could be attributed to the closed counterpressure chamber and the resulting partially prevented contact of the melt with the atmospheric oxygen, taking into account that the device is sealed against the melt but not necessarily against the atmospheric oxygen. In addition, in any case, thermal degradation of the material would also have occurred over the long test period of about 10 h, which should also be reflected in a lower viscosity number.

Therefore, it is assumed that the almost permanent pressurization of the melt restricts the chain mobility to such an extent that no thermally vibrational induced degradation of the molecular bonds can take place. A pressure-induced restriction of the chain mobility would inevitably also lead to a reduced oxygen diffusion rate and, thus, also hinder thermo-oxidative damage. This is the subject of the current investigations on the pressure-dependent degradation of thermoplastic melts at the Institute of Polymer Technology.

**Figure 9:**
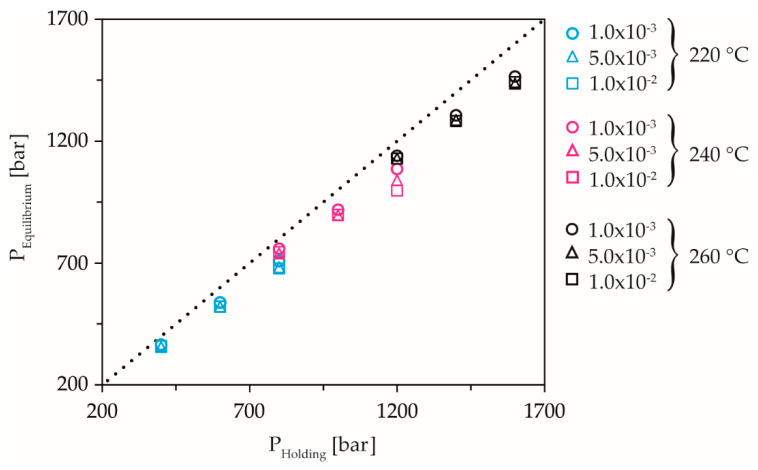
Equilibrium pressure as a function of holding pressure, temperature, and compression rate.

The proof of the elastic melt compression in the measuring chamber after dynamic solidification can be provided by the comparison between the holding pressure and the equilibrium pressure in Figure 9.

The equilibrium pressure is the pressure after completion of the pressure equalization processes, thus, the pressure at which the signals intersect. At low holding pressure levels, the holding pressure and equilibrium pressure hardly differ from each other, indicating that no elastic compression of the melt has taken place. At higher compression rates, there is already a deviation between holding pressure and equilibrium pressure, with the highest holding pressure and the highest compression rate showing the greatest difference to the equilibrium pressure.

**Figure 10:**
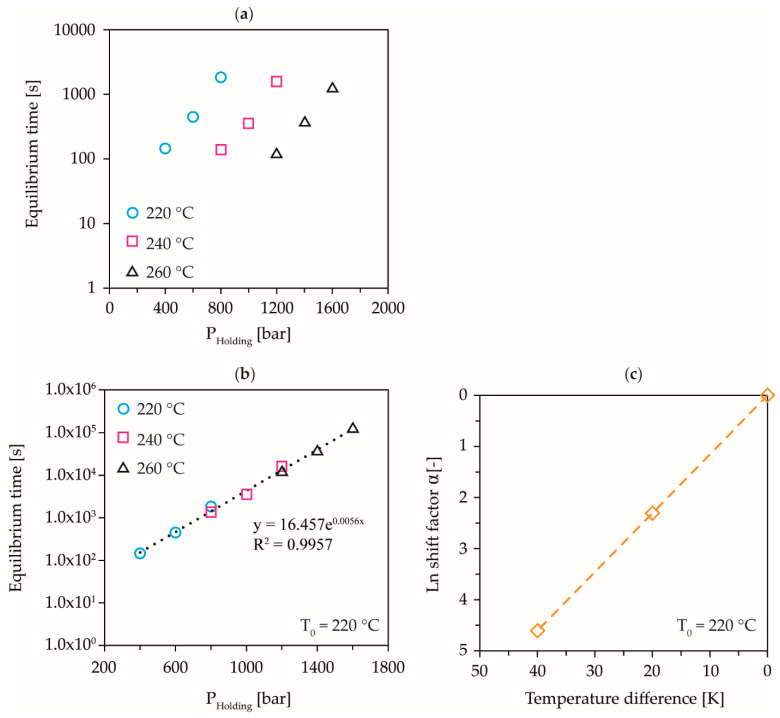
Construction of the master curve to calculate the equilibrium time, as a function of the holding pressure and the temperature at a compression rate of 1.0 × 10^−3^; logarithmic plot of the measured equilibrium times (**a**), shift of the measured equilibrium times to a master curve at 220 °C and 1.0 × 10^−3^ (**b**), and temperature shift factors (**c**).

With the measuring method shown, the determination of the viscous equilibrium time, which is needed to complete all flow processes during the holding pressure phase, as a function of pressure and temperature under process-relevant conditions, seems to be possible. The knowledge of the relationship between pressure, temperature, compression rate, and the resulting flow processes during the holding pressure phase is essential for the exact calculation of the formation of molecular orientations in the injection molding process, since here, high-pressures can act in addition to high temperature gradients. In the holding pressure phase of the injection molding process, the melt is compressed within a completely filled mold over a narrow flow cross-section, at a constant compression rate to a specific pressure level. In the selected measuring setup, the mold corresponds to the filled CPC, the solidifying narrow gate corresponds to the capillary and the screw antechamber corresponds to the measuring chamber. Thus, the described measuring setup allows the simulation of the holding pressure phase of the injection molding process, under laboratory conditions. If the measured equilibrium times are plotted logarithmically, as a function of the holding pressure, a temperature-invariant behavior is shown, which allows the measurement results of the test setup shown here to be generalized, Figure 10a. The shift of the individual equilibrium curves by a temperature shift factor allows the derivation of a generalized relationship for the calculation of the so defined viscous pressure equilibrium time, as a function of temperature and holding pressure, Figure 10b,c. Due to the compression-rate-dependence of the equilibrium times, this is only allowed for one defined compression rate.

Figure 10 shows the displacement of the individual measuring points to a master curve using the compression rate 1.0 × 10^−3^ and the reference temperature 220 °C as an example. The temperature invariant plotting allows the exponential fit of the curve to derive an equation for the calculation of the temperature and pressure-dependent equilibrium time. Table 3 summarizes the derived master curves as a function of the examined compression rates:

## 4. Conclusions

The use of integrative simulation techniques to predict internal component properties, such as residual stresses and orientations, which can influence the optical, mechanical, and shrinkage properties of injection molded components, is becoming increasingly important. For a reliable prediction of process-induced orientations during injection molding, a characterization of the pressure, temperature, and compression-rate-dependent flow behavior during the holding pressure phase is necessary. This study shows the attempt to simulate the flow behavior in the holding pressure phase of the injection molding process, under laboratory conditions with a high-pressure capillary rheometer and a closed counterpressure chamber. Previous investigations with this measuring setup could reveal a compression rate-dependent melt solidification under unsteady flow conditions. In order to characterize the equalization behavior of dynamically solidified and still flowable material under transient flow conditions, the dynamic solidification behavior of a cyclic-olefin copolymer relevant for optical applications was characterized and a corresponding experimental design for the equilibrium investigations was derived. These investigations show an exponential increase of the pressure equilibrium time, with increasing holding pressure level and a decrease of the equilibrium time with increasing temperature. In addition, a compression-rate-dependence of the equilibrium times is shown at high compression rates in the area of dynamic melt solidification. The measurements also show a temperature invariant behavior, which allows the derivation of a master curve for the calculation of the equilibrium times as a function of temperature and holding pressure for an underlying compression rate. With the help of the empirically determined equations, the temperature-, pressure-, and compression-rate-dependent stop of the flow, as well as time-dependent reflow effects, can be modelled for consideration in integrative simulations to improve the prediction of molecular orientations.

## Figures and Tables

**Table 1:** Experiments to determine the dynamic pressure-induced solidification of the COC.

Compression Rate [1/s]	Temperature [°C]
1.8 × 10^−5^	210
	220
	230
	240
	250
1.8 × 10^−4^	210
	220
	230
	240
	250
3.5 × 10^−4^	210
	220
	230
	240
	250

**Table 2:** Experiments to determine the pressure-dependent equilibrium time of the COC; all pressures were varied at each temperature and compression rate.

Temperature [°C]	Compression Rate [1/s]	Holding Pressure [bar]
220	1.0 × 10^−3^	400
	5.0 × 10^−3^	600
	1.0 × 10^−2^	800
240	1.0 × 10^−3^	800
	5.0 × 10^−3^	1000
	1.0 × 10^−2^	1200
260	1.0 × 10^−3^	1200
	5.0 × 10^−3^	1400
	1.0 × 10^−2^	1600

**Table 3:** Derived master curves to calculate the pressure and temperature dependent equilibrium time of the COC for all investigated compression rates.

Compression Rate [1/s]	Master Curve Equation	Shift Factor α [-]
1.0 × 10^−3^	$t_{eq}=exp\left(0.0056\times P_{Holding}+\alpha \times \Delta T+2.80\right)$	−0.11
5.0 × 10^−3^	$t_{eq}=exp\left(0.0054\times P_{Holding}+\alpha \times \Delta T+2.82\right)$	
1.0 × 10^−2^	$t_{eq}=exp\left(0.0054\times P_{Holding}+\alpha \times \Delta T+2.85\right)$	

## Data Availability

The data presented in this study are available on request from the corresponding author.
